# Comparative Cytochrome P450 *In Vitro* Inhibition by Atypical Antipsychotic Drugs

**DOI:** 10.1155/2013/792456

**Published:** 2013-02-13

**Authors:** Guillermo Gervasini, Maria J. Caballero, Juan A. Carrillo, Julio Benitez

**Affiliations:** Department of Medical and Surgical Therapeutics, Division of Pharmacology, Medical School, University of Extremadura, Av. Elvas s/n, 06071 Badajoz, Spain

## Abstract

The goal of this study was to assess in human liver microsomes the inhibitory capacity of commonly used antipsychotics on the most prominent CYP450 drug metabolizing enzymes (CYP1A2, CYP2C9, CYP2D6, and CYP3A). Chlorpromazine was the only antipsychotic that inhibited CYP1A2 activity (IC_50_ = 9.5 **μ**M), whilst levomepromazine, chlorpromazine, and thioridazine significantly decreased CYP2D6-mediated formation of 1′-hydroxybufuralol (IC_50_ range, 3.5–25.5 **μ**M). Olanzapine inhibited CYP3A-catalyzed production of 1′, and 4′-hydroxymidazolam (IC_50_ = 14.65 and 42.20 **μ**M, resp.). In contrast, risperidone (IC_50_ = 20.7 **μ**M) and levomepromazine (IC_50_ = 30 **μ**M) showed selectivity towards the inhibition of midazolam 1′-hydroxylation reaction, and haloperidol did so towards 4′-hydroxylation (IC_50_ of 2.76 **μ**M). Thioridazine displayed a *K*_*i*_ of 1.75 **μ**M and an inhibitory potency of 1.57 on CYP2D6, suggesting a potential to induce *in vivo* interactions. However, with this exception, and given the observed *K*_*i*_ values, the potential of the assayed antipsychotics to produce clinically significant inhibitions of CYP450 isoforms *in vivo* seems limited.

## 1. Introduction

In contrast to conventional neuroleptics, atypical antipsychotics have been shown to be effective against both positive and negative symptoms of schizophrenia while showing a reduced propensity to induce extrapyramidal effects [1]. The cytochrome P450 (CYP) 1A2, CYP2C9, CYP2D6, and CYP3A enzymes are responsible for the metabolism of many of these and other psychoactive compounds [2–8]. Traditionally, studies on pharmacological interactions involving antipsychotics have examined the inhibition of these isoforms by drugs that are concomitantly administered, especially serotonin reuptake inhibitors [9–13]. However, there is still limited information on the capacity of antipsychotics to inhibit the metabolism of other coadministered drugs and the potentially clinically significant interactions that could arise [14].

In this paper we have assessed the potential for nine of the most commonly used atypical antipsychotics (clozapine, olanzapine, iloperidone, quetiapine, haloperidol, chlorpromazine, levomepromazine, thioridazine, and risperidone) and abaperidone (7-[3-[4-(6-fluoro-1,2-benzisoxazol-3-yl)-piperidin-1-yl]propoxy]-3-(hydroxymethyl)chromen-4-one; FI-8602), an underdevelopment compound with a potentially atypical antipsychotic profile [15, 16], to cause significant drug-drug interactions by using *in vitro* inhibition techniques in human liver microsomes.

## 2. Methods

### 2.1. Reference Substances

Abaperidone and iloperidone were gifts from Centro de Investigación Farmacéutica, Grupo Ferrer (Barcelona, Spain). Chlorpromazine, levomepromazine, thioridazine, haloperidol, risperidone, clozapine, quinidine, sulphaphenazole, ketoconazole, diclofenac, furafylline, phenacetine, and acetaminophen were purchased from Sigma Chemical Co. (Madrid, Spain). Midazolam, 1^'^-hydroxy-midazolam, and 4-hydroxymidazolam were supplied by Hoffman-La-Roche (Basel, Switzerland). Bufuralol and 1′-hydroxybufuralol were obtained from Ultrafine Chemicals (Manchester, England), 4-hydroxydiclofenac from Gentest Corp (Woburn, MA), -quetiapine from Astra Zeneca (Macclesfield, Chesire, UK), and olanzapine from Lilly Laboratories (Indianapolis, IN).

### 2.2. Chemicals

Methanol and acetonitrile were of HPLC grade (Merck, Barcelona, Spain). Acetic and formic acids were of analytical grade and supplied by Merck (Barcelona, Spain). Glucose-6-phosphatedehydrogenase, glucose-6-phosphate, and NADPH were purchased from Roche Diagnostics SL (Barcelona, Spain). All other chemicals and reagents used were of the highest commercially available quality. Water was filtered by a Milli Q water system from Millipore Ibérica, S.A. (Madrid, Spain).

### 2.3. Chromatography

Chromatographic separation was performed with a system consisting of a Hewlett-Packard (HP) 1100 series (Agilent Technologies Spain S.L., Madrid, Spain) that had a degasser (model G1322A), quaternary pump (model G1311A), autosampler (model G1313A), column oven compartment (model G1316A), ultraviolet/visible detector (model G1315A), and fluorescence detector (model G and a mass spectrometer engine (model G1946A). A computer-assisted HP G2710AA LC/MS ChemStation (Agilent Technologies Spain S.L., Madrid, Spain) was used to operate modules and facilitate data management.

Phenacetin and its metabolite paracetamol were separated on a Hypersil BDS C18, 3 *μ*m particle size, 100 cm × 3.0 mm reversed-phase column (Agilent Technologies Spain S.L., Madrid, Spain). The composition of the mobile phase was 18% acetonitrile: 82% acetic acid (0.1%) (vol/vol). The flow rate through the column at 30°C was 0.3 mL/minute and phenacetin and its oxidized metabolite paracetamol were monitored by ultraviolet absorbance at 247 nm.

Bufuralol and its metabolite 1′-hydroxybufuralol were separated on an Ultrasphere ODS, 3 *μ*m particle size, 7.5 cm × 4.6 mm reversed-phase column (Beckman Instruments, Madrid, Spain). The composition of the mobile phase was 40% acetonitrile: 60% acetic acid (0.1%) (vol/vol). The flow rate through the column at 30°C was 1 mL/minute. Bufuralol and its oxidized metabolite 1′-hydroxybufuralol were monitored by fluorescence detection (excitation at 252 nm; emission at 302 nm).

Diclofenac and its metabolite 4′-hydroxydiclofenac were separated on a Hypersil BDS C18, 3 *μ*m particle size, 100 cm × 3.0 mm reversed-phase column (Agilent Technologies Spain S.L., Madrid, Spain). The composition of the mobile phase was 50% acetonitrile : 50% acetic acid (0.1%) (vol/vol). The flow rate through the column at 30°C was 0.4 mL/minute, and diclofenac and its oxidized metabolite 4′-hydroxydiclofenac were monitored by ultraviolet absorbance at 220 nm Midazolam, 4-hydroxymidazolam and 1′-hydroxymidazolam were separated on an Ultrasphere ODS, 3 *μ*m particle size, 7.5 cm × 4.6 mm reversed-phase column (Beckman Instruments, Madrid, Spain). The composition of the mobile phase was 39% acetonitrile : 61% formic acid (0.1%) (vol/vol). The flow rate through the column at 30°C was 0.5 mL/minute and midazolam, 4-hydroxymidazolam, and 1′-hydroxymidazolam were monitored by means of an HPLC/electrospray ionization-mass spectrometry (LC/ESI-MS) method with isocratic elution.

The mass spectrometer was run in the positive ion mode. Nitrogen as the drying gas was supplied at a flow of 10 L/minutes and at a temperature of 350°C. The capillary voltage was adjusted to 4100 V. To quantitate midazolam, 4-hydroxymidazolam, and 1′-hydroxymidazolam, the mass spectrometer was operated in the selected ion-monitoring mode (SIM) with a dwell time of 229 msec. The eluant from HPLC was introduced into the source via the electrospray interface, generating the positively charged pseudomolecular ions (MH+) at m/z 326 and 342.

### 2.4. Inhibition Studies

Inhibition studies on four different CYP isoform-specific substrates were carried out in human liver microsomes (Gentest Corporation, Woburn, MA). Assessed reactions were phenacetin O-deethylation for CYP1A2, (+/−)-bufuralol 1′-hydroxylation for CYP2D6, diclofenac 4′-hydroxylation for CYP2C9, and midazolam 1′- and 4′-hydroxylation for CYP3A. The different antipsychotics were dissolved in methanol and serially diluted with 100 mM tris buffer (pH = 7.5) to obtain final concentrations ranging from 0.05 to 50 *μ*M. Abaperidone was an exception and was dissolved in hot water up to a maximum concentration of 20 *μ*M.

The conditions of the incubation reactions as well as the concentration of the substrate reaction probes utilized were established based on previous CYP450 inhibition studies by our group in human liver microsomes [20–24]. In brief, a 0.25 mL reaction mixture consisting of 0.8 mg/mL microsomal protein, a NADPH regenerating system, and the specific reaction substrate at a concentration equivalent to its Km value (100 *μ*M phenacetin, 50 *μ*M bufuralol, 15 *μ*M diclofenac, or 10 *μ*M midazolam) was incubated in the absence or presence of increasing concentrations of the tested antipsychotics. Reactions were started by adding microsomal protein and the mix was then vortexed and incubated at 37°C for 30 min. The process was stopped by the addition of acetonitrile and incubation on ice during 10 minutes. Samples were then centrifuged at 10,000 g for 3 minutes, and the supernatant was taken for subsequent HPLC analysis. Control inhibition studies were carried out with prototypic inhibitors in increasing concentrations up to 20 *μ*M, namely, furafylline (CYP1A2), quinidine (CYP2D6), sulphaphenazole (CYP2C9), and ketoconazole (CYP3A).

The effect of antipsychotics on the rate of CYP-catalyzed probe substrates was expressed as a percentage of the control activity with no antipsychotic present. The concentration of inhibitor causing 50% reduction of activity relative to the appropriate control value (IC_50_) was calculated by interpolation of a regression line of the log concentration versus percent inhibition plot over at least three concentrations of inhibitor. *K*_*i*_  estimates were obtained as described previously [17].

## 3. Results and Discussion

The chromatographic conditions efficiently separated the compounds of interest in all the assays. Calibration curves were linear (coefficients of correlation >0.99) within the range of concentrations established (0.5 to 100 *μ*M for phenacetin and bufuralol assays, 1–25 *μ*M for diclofenac, and 0.2 to 20 *μ*M for midazolam). The retention times (given in minutes) for the analyzed substances were as follows: paracetamol: 2.3; phenacetin: 11.2; 1′-hydroxybufuralol: 4.6; bufuralol: 27; 4′-hydroxydiclofenac: 2.5; diclofenac: 5.4; 4′-hydroxymidazolam: 4.9; 1′-hydroxymidazolam: 5.8; midazolam: 8.7.

**Figure 1:**
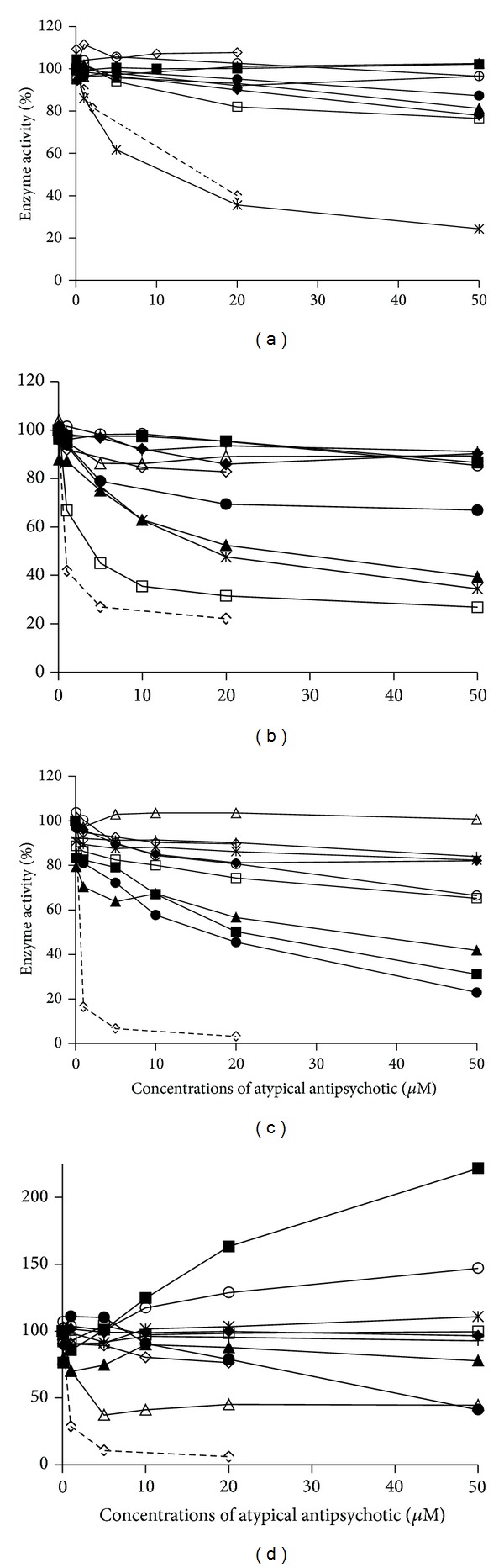
Inhibition of CYP1A2-mediated phenacetin O-deethylation (a), CYP2D6-mediated bufurarol 1-hydroxylation (b), and CYP3A-mediated midazolam 1′- (c) and 4′-hydroxylation (d) by atypical antipsychotics. Drugs depicted are clozapine (♦), olanzapine (●), iloperidone (+), quetiapine (○), haloperidol (∆), chlorpromazine (x), levomepromazine (▲), thioridazine (□), risperidone (■), and abaperidone (*◊*). Dotted line denotes the corresponding prototypic ihhibitor.

The incubation of 100 *μ*M phenacetin with multiple antipsychotic concentrations showed that only chlorpromazine was an inhibitor of CYP1A2 activity (IC_50_ = 9.5 *μ*M; Figure 1(a)). In line with this, a recent study have suggested that pharmacokinetic interactions involving chlorpromazine and CYP1A2 substrates are likely to occur, as chlorpromazine biotransformation exhibits a stricter CYP1A2 preference compared to other phenothiazines [25].

Levomepromazine, chlorpromazine, and thioridazine were significant inhibitors of CYP2D6, showing IC_50_ values of 25.5, 20, and 3.5 *μ*M, respectively (Figure 1(b)). In this regard, Shin et al. have previously shown that chlorpromazine and thioridazine were able to significantly inhibit CYP2D6 [26], albeit the authors also reported an inhibitory effect of risperidone, clozapine, and haloperidol, which we did not observe under our experimental conditions.

We could not identify any inhibitors of the CYP2C9 activity under our experimental conditions.

The observed CYP3A inhibition rates were different depending on the measured metabolite. Thus, olanzapine (IC_50_ = 14.65 *μ*M), risperidone (IC_50_ = 20.7 *μ*M), and levomepromazine (IC_50_ = 30 *μ*M) inhibited midazolam 1′-hydroxylation (Figure 1(c)). Eap et al. have previously argued against risperidone being an inhibitor of CYP450 [27], but it should be noted that the authors did not assess CYP3A activity. The other CYP3A-mediated reaction, midazolam 4′-hydroxylation, was inhibited by both olanzapine (IC_50_ = 42.20 *μ*M) and haloperidol (IC_50_ = 2.76 *μ*M) (Figure 1(d)).

Given the *K*_*i*_ estimates presented in Table 1, the most likely antipsychotic to produce an *in vivo* inhibition of a CYP enzyme would be thioridazine on CYP2D6. Indeed, our group has previously reported that subjects taking thioridazine were more prone to display a CYP2D6 poor metabolizer phenotype [28]. *K*_*i*_ values obtained for the other antipsychotics do not allow for anticipating a significant effect *in vivo* (Table 1). However, there are a number of reports of *in vivo* interactions caused by these agents; for example, risperidone and olanzapine have been shown to modify the pharmacokinetics of aripiprazole, another antipsychotic metabolized by CYP450 isoforms [29]. A possible explanation for this fact would be the existence of drug concentrations in the liver far superior to those in blood [30, 31] or inhibitions due to drug metabolites [32].

Interestingly, abaperidone did not cause a significant inhibition of any of the CYP enzymes assayed. This is a pharmacologically active substance underdevelopment, which therefore appears to be a remarkably safe agent with regard to CYP-mediated drug interactions, the main mechanism underlying interactions involving atypical antipsychotics [33, 34]. It should be noted, however, that certain antipsychotics may also interact with other drugs via P-glycoprotein inhibition [35], and further studies are warranted to evaluate the effect of this drug on this and other ABC transporters.

In summary, the results show that several commonly used atypical antipsychotics exerted a significant *in vitro* inhibitory capacity of CYP450 enzymes. However, and with the exception of thioridazine, the potential to cause significant *in vivo* interactions appears to be limited.

## Figures and Tables

**Table 1:** Inhibitory effect of atypical antipsychotics on human liver microsomal CYP450 activity.

Antipsychotic	*I*_max⁡_ (%)	^ a^ *K*_*i*_ (*μ*M)	^ b^Therapeutic range (*μ*M)	^ c^Inhibitory potency
CYP1A2				
Chlorpromazine	75.7	4.75	0.1–1	0.1158
CYP2D6				
Levomepromazine	60.6	12.75	0.05–0.2	0.0098
Chlorpromazine	65.5	10.0	0.1–1	0.0550
Thioridazine	73.2	1.75	0.5–5	1.5714
CYP3A (1-hydroxylation)				
Olanzapine	77.1	7.30	0.06–0.25	0.0212
Risperidone	70.0	10.35	0.04–0.15	0.0092
Levomepromazine	58.2	15.0	0.05–0.2	0.0083
CYP3A (4′-hydroxylation)				
Olanzapine	58.8	21.1	0.06–0.25	0.0073
Haloperidol	55.4	1.38	0.01–0.04	0.0181

*I*_max⁡_, maximum inhibition.

^a^ *K*_*i*_ estimates were obtained as described previously [17].

^b^Retrieved from Kirchherr and Kühn-Velten [18].

^c^Intrinsic inhibitory potency ([inhibitor]/*K*_*i*_), a measure of the potency of an inhibitor [19], was calculated relative to the mean of reported therapeutic concentrations [18].
